# Microscopic Video-Based Grouped Embryo Segmentation: A Deep Learning Approach

**DOI:** 10.7759/cureus.45429

**Published:** 2023-09-17

**Authors:** Huy Phuong Tran, Hoang Thi Diem Tuyet, Truong Quang Dang Khoa, Le Nhi Lam Thuy, Pham The Bao, Vu Ngoc Thanh Sang

**Affiliations:** 1 Department of Infertility, Hung Vuong Hospital, Ho Chi Minh City, VNM; 2 Faculty of Engineering and Technology, Mekong University, Vinh Long, VNM; 3 IC-IP Lab, Faculty of Information and Technology, Saigon University, Ho Chi Minh City, VNM

**Keywords:** microscopic video processing, in vitro fertilization, blur detection, segmented image organization, embryo segmentation

## Abstract

Purpose: The primary aim of this research is to enhance the utilization of advanced deep learning (DL) techniques in the domain of in vitro fertilization (IVF) by presenting a more refined approach to the segmentation and organization of microscopic embryos. This study also seeks to establish a comprehensive embryo database that can be employed for future research and educational purposes.

Methods: This study introduces an advanced methodology for embryo segmentation and organization using DL. The approach comprises three primary steps: Embryo Segmentation Model, Segmented Embryo Image Organization, and Clear and Blur Image Classification. The proposed approach was rigorously evaluated on a sample of 5182 embryos extracted from 362 microscopic embryo videos.

Results: The study’s results show that the proposed method is highly effective in accurately segmenting and organizing embryo images. This is evidenced by the high mean average precision values of 1.0 at an intersection over union threshold of 0.5 and across the range of 0.5 to 0.95, indicating a robust object detection capability that is vital in the IVF process. Segmentation of images based on various factors such as the day of development, patient, growth medium, and embryo facilitates easy comparison and identification of potential issues. Finally, appropriate threshold values for clear and blur image classification are proposed.

Conclusion: The suggested technique represents an indispensable stage of data preparation for IVF training and education. Furthermore, this study provides a solid foundation for future research and adoption of DL in IVF, which is expected to have a significant positive impact on IVF outcomes.

## Introduction

As advancements in reproductive medicine continue to evolve, the quest for more effective and reliable methods of in vitro fertilization (IVF) remains a focal point of research and clinical practice. The journey from fertilization to a viable embryo involves a myriad of biological processes, each sensitive to a range of environmental factors. Accurate and non-intrusive monitoring of these processes is crucial for the success of IVF treatments. Traditional methods have provided some insights but come with their own sets of limitations. The emergence of new technologies and computational methods offers promising avenues for overcoming these challenges, thereby potentially increasing the success rates of IVF procedures. Embryo culture in IVF is a carefully orchestrated procedure designed to mimic, as closely as possible, the natural conditions that allow an embryo to develop. While solitary culture, the practice of culturing each embryo in its separate drop of culture medium, has its merits, the alternative approach of group culturing is garnering interest within the scientific and medical communities. In group culturing, multiple embryos are placed together in a single droplet of culture medium, offering a unique microenvironment that can improve developmental outcomes [1].

In many clinical settings, microscopic evaluation remains the standard method for assessing embryo development during IVF. This approach involves scheduled observations under a microscope to assess key developmental markers such as morphology and cell division. However, this technique often requires removing the embryo from its controlled culture environment for brief periods, which poses some risks. Time-lapse technology is emerging as an alternative that allows for continuous, non-intrusive monitoring within the incubator, capturing detailed and dynamic information on embryonic development. While this technology has been gaining traction in developed countries due to its advantages, its adoption in developing countries may be limited by factors such as cost and availability of advanced infrastructure. Both methods have their own sets of advantages and challenges, and the choice between them may depend on a variety of considerations including resources, expertise, and specific clinical needs.

The shift from conventional microscopic evaluation to automated systems using artificial intelligence (AI) in IVF is advancing through several key stages. Firstly, the extensive dataset is created using either manual efforts or segmentation techniques, leading to the precise isolation of the embryo from its surroundings. Following this segmentation, the segmented images are organized into separate folders based on predefined criteria, and they undergo meticulous labeling. Finally, the deep learning (DL) model is trained for embryo assessment based on predefined medical criteria.

This research signifies the initial advancement in shifting away from conventional embryo evaluation approaches toward the cutting-edge domain of DL-facilitated embryo assessment. In the context of this investigation, the focus is on embryos cultured in pairs, which presents a significant challenge. Consequently, the input dataset encompasses microscopic videos capturing this dynamic embryo development. The ultimate goal is to achieve a precise segmentation and well-organization of these embryos as the desired output. This preliminary phase establishes the groundwork for subsequent DL endeavors that seek to enhance and progress embryo evaluation techniques.

Literature review

Embryo segmentation is vital in evaluating embryo development using DL because it permits the extraction of key morphological aspects that can influence embryo maturation and viability. It involves identifying and separating regions of interest (ROI) from microscopic images or videos. Accurate segmentation of embryos is critical for downstream analyses, such as embryo tracking and evaluation. Conventional segmentation methods have limitations due to variations in embryo morphology and imaging conditions. Recently, numerous techniques have been suggested to tackle this challenge. This literature review offers a comprehensive overview of the latest advancements in embryo-partitioning approaches, concentrating on the segmentation of distinct embryo features at different phases of development.

In the investigation [2], the authors evaluated the precision and reliability of measurements obtained through a convolutional neural network (CNN) concerning the segmentation of zygote cytoplasm. This assessment involved a comparison with evaluations performed by proficient embryologists. They developed an automated system designed for the segmentation of human zygote cytoplasm. This system employed images and harnessed the capabilities of a CNN model that was trained on a dataset containing 550 zygote images, each meticulously annotated by embryologists. Impressively, the CNN achieved an Intersection over union (IoU) score of 96.15 ± 2.28%. IoU is a metric used to evaluate the accuracy of an object detector on a particular dataset; it measures the overlap between the predicted segmentation area and the ground truth, divided by the union of both areas. In this case, the high IoU score indicates that the model was highly successful in segmenting day-1 embryos, even when accounting for variations in shape, background noise, and luminance.

Another study aims to assess the efficacy of automated image segmentation techniques for human embryos on day 5, particularly those in the blastocyst stage [3]. The focus is on the U-Net architecture and its variants. The investigation utilizes a proprietary dataset comprising 258 images of blastocyst-stage embryos collected from Acıbadem Fulya Hospital’s IVF Center and captured through the Embryoscope system. In addition, a publicly available dataset containing 249 embryo images is also incorporated. The customized Dilated Inception U-Net model demonstrated exceptional performance in the study. U-Net is a type of CNN specifically designed for biomedical image segmentation, and the Dilated Inception modification enhances its ability to capture contextual information. The model achieved a Dice coefficient of 98.68%, a metric that quantifies the similarity between the predicted and ground truth segmentation by calculating twice the area of overlap divided by the sum of the areas of both sets. Additionally, the model scored a Jaccard index of 97.52%, another measure of similarity that evaluates the intersection over the union of the predicted and actual segmented areas. These high scores indicate that the model is extremely accurate in segmenting biomedical images, validating its efficacy for the task at hand.

The approach employed in the study involves the segmentation of embryos within video frames and the prediction of the frame where cell cleavage initiates [4]. Furthermore, the method successfully identified morphological features associated with subsequent stages of cell cleavage, including morula and blastocyst. The dataset used in the study encompassed 250 embryo videos, comprising embryos used for female transfers as well as those preserved through cryopreservation for future utilization. The study goes into detail about the training process for two specialized tools, YOLO v5 and DETR, which are used for identifying and locating specific objects within images. YOLO v5, which stands for You Only Look Once version 5, is a highly efficient tool that scans an image in a single pass to identify objects. DETR, or DEtection TRansformer, is another tool that uses a different approach but aims for the same goal as accurate object detection. Notably, YOLO v5 achieved what’s known as mean average precision (mAP) scores of 0.65, 0.78, and 0.80 when detecting cells, morulae, and blastocysts, respectively. In simpler terms, these scores are like grades; the closer they are to 1, the better the tool is at accurately identifying these specific objects in images.

Prior efforts have predominantly revolved around analyzing solitary embryos per frame, streamlining data collection, and simplifying subsequent analyses. In contrast, the current study addresses the intricacies inherent to real-world scenarios by introducing a paradigm shift: the analysis of two embryos per frame. This adjustment acknowledges the complexity of interactions within a group setting, presenting a more realistic representation of embryonic development.

An equally notable contribution is observed in terms of data sourcing. While traditional methodologies heavily rely on static still images, offering ease of analysis but lacking the ability to capture the dynamic temporal aspects of embryonic processes, the current study leverages video frames. This dynamic data source introduces a temporal dimension to the analysis, capturing the evolving nature of embryonic behaviors. It should be noted that the decision to work with video frames introduces computational demands.

Prior studies often operated in controlled settings with minimal noise interference. In contrast, the present methodology takes an adaptive approach, equipped to manage irregularities such as blurred and transitional frames. This adaptability broadens the applicability of the research, making it well-suited for real-world scenarios where such challenges are prevalent. However, the adaptability also introduces complexities, necessitating advanced algorithms to navigate the intricacies of noise and complexity effectively.

The research also distinguishes itself in terms of automation strategies. While previous approaches often relied on semi-automated techniques that might involve manual interventions, the current study is intrinsically geared toward full automation. This orientation aligns with the demand for efficiency and scalability, making it an ideal fit for broader, less-controlled environments where manual interventions might be impractical. 

Furthermore, the research extends its contribution potential beyond its current phase. The inherent complexity of the study’s approach opens avenues for subsequent exploratory endeavors. The preliminary nature of the research highlights its developing stage, while the comparative analysis underscores its potential to advance the field significantly. Nevertheless, it remains crucial to acknowledge and address the challenges that arise, emphasizing the necessity for further in-depth investigation and innovation.

For an in-depth review of object segmentation employing DL techniques, readers are recommended to refer to other papers [5,6]. This research predominantly focuses on segmenting the complete embryo, which is why the segmentation of specific embryo components falls beyond the scope of this study. Furthermore, individuals interested in exploring intricate segmentation of embryo components are recommended to consult relevant literature concerning day 1 [7-9] and day 5 [10-12].

## Materials and methods

An in-depth examination of existing literature and methodologies reveals a clear contribution of this research to the field, as presented in Table 1.

**Table 1:** Comparison between the related works and this work

Criteria	Related Works [2–4]	This Work
Subject(s)	Single embryo per frame	Two embryos per frame
Data Source	Still images [2,3] and videos [4]	Video
Noise Handling	No irrelevant or noise frames	Contains blur and transition frames
Advantages	High accuracy due to clean data	Capable of handling real-world, noisy data; more comprehensive analysis
Challenges	Limited to ideal conditions; may require manual selection	Requires advanced algorithms to handle noise and complexity; still in the preliminary phase
Real-World Applicability	May not handle real-world complexities	Designed for real-world scenarios
Potential for Future Research	Limited scope for extension	High potential for further advancements

Problem definition

**Figure 1:**
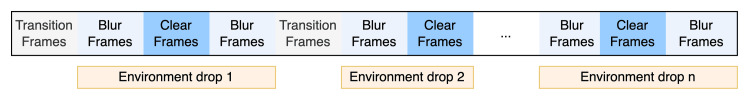
Sequence of frames in the embryo evaluation process

The research presented here addresses a compelling challenge in current procedures toward the adopting AI-based technologies. Figure 1 describes the sequence of frames in the embryo evaluation process. Each input consists of an observation video on grouped embryos in several environment drops. A single environment drop includes a sequence of frames: first, a set of blurry frames because the embryologist is adjusting the camera’s focus, followed by a series of clear frames for evaluation purposes, and finally, additional blurry frames as the camera transitions to the next environment. After a complete evaluation of one environmental drop, the embryologist adjusts the microscope to transition to the next environmental drop, resulting in the generation of a blurred frame between observations of each environmental drop. 

The primary focus of this study is to develop a comprehensive, fully automated DL-based methodology for the accurate segmentation, organization, and classification of embryos in microscopic videos. The problem statement involves processing video frames as input and generating organized folders containing distinct, clear images of individual embryos as output. This procedure is broken down into three critical stages.

The first stage is "Embryo Segmentation", which involves isolating grouped embryos from clear frame sequences. This stage leverages DL models capable of handling multiple embryos per frame and real-world complexities like noise and blur. The second stage, "Embryo Organization", entails meticulously arranging the segmented embryo images into their respective folders based on predefined criteria. This process facilitates easier data management and subsequent analysis. In this stage, each environmental drop contains two embryos, which should be organized into separate folders. The final stage is "Image Classification" within the organized folders, which involves the selection and classification of clear embryo images while removing any blurred or otherwise compromised images. This step ensures the highest quality data for further research and clinical applications.

Data description

The dataset comprises 176 videos of 2928 day-3 embryos and 186 videos of 2254 day-5 embryos, captured at 25 frames per second using an optical inverted microscope. However, to reduce the computational workload and avoid redundancy, the input frames are downsampled to five frames per second. Several day-3 embryos may not survive to day 5, leading to a difference in the number of videos and images between the two groups. The images and videos were acquired using consistent imaging conditions, ensuring consistent image quality and resolution. This dataset provides a valuable resource for researchers and clinicians in IVF and embryonic development. The process of collecting embryo data and conducting embryo segmentation was conducted in accordance with the approved guidelines and protocols of the local ethics committee.

**Figure 2:**
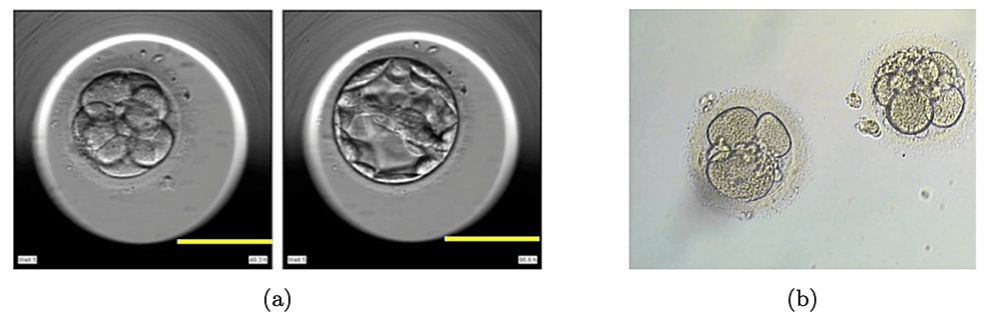
Sample clear frames from the related work (a) and this work (b) Adapted from [4].

Figure 2 presents exemplar frames from both the related work and the current study. The preceding research emphasizes sharply focused objects within the data frames, excluding any images with a blue hue or transitional content. Furthermore, each image in the prior study contains a singular object. In contrast, our dataset encompasses videos with a mix of clear, blurred, and transitional images. Within the clear images, frames feature two embryos, posing an additional layer of complexity for object segmentation.

**Figure 3:**
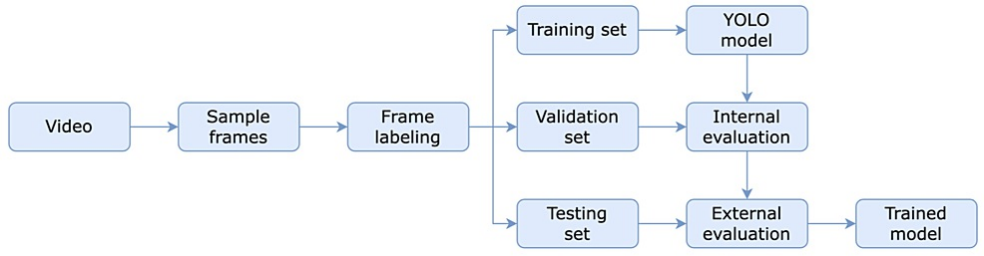
Overview of the embryo segmentation process YOLO: You Only Look Once

The flowchart in Figure 3 depicts a multi-stage process to create a YOLO model to segment embryos in a frame [13]. The process begins with acquiring video data, which is then partitioned into individual frames. These frames are then labeled with annotations corresponding to the embryos depicted by an embryologist. The labeled frames are divided into three sets: training (180 images), validation (60 images), and testing (60 images). This division ensures that the model is trained and tested on non-overlapping data, which improves the model generalization. The training set is then used to train the YOLO model. The YOLO model is designed to identify embryos in the entire image simultaneously, enabling fast and efficient processing. Once the model has been trained, its performance is evaluated internally and externally. The external evaluation measures the model's performance on the training set, while the external evaluation assesses its ability to generalize to new data. mAP is used to quantify the model’s performance. Finally, the model is applied to the testing set to detect objects in independent scenarios, and the trained model is generated. This model is deployed in a later process. 

Segmented embryo image organization

**Figure 4:**
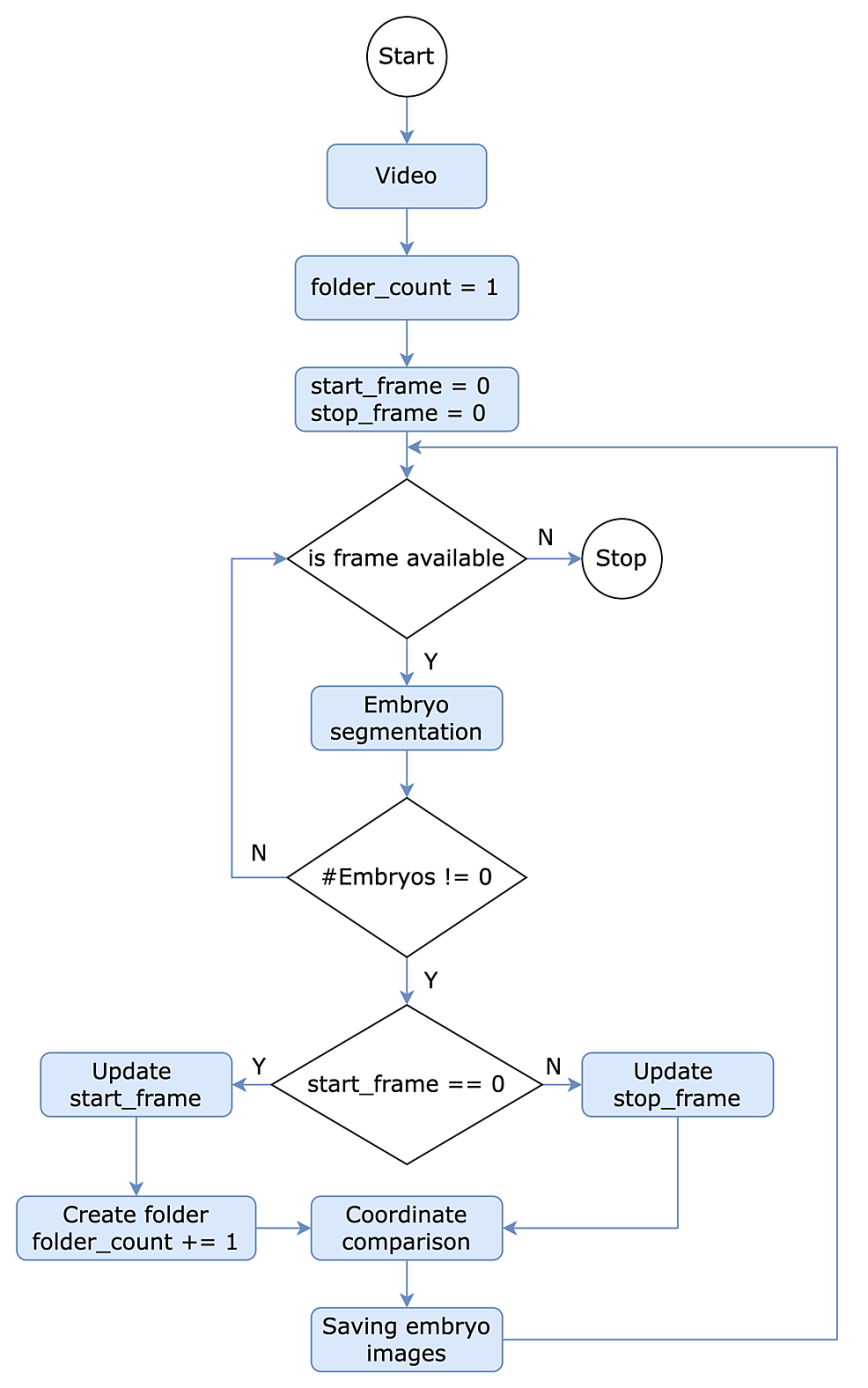
Overview of the embryo organization process

Figure 4 outlines an intricate and comprehensive procedure for processing video frames and meticulously extracting segmented embryo images, ultimately organizing them. The detailed and complex process involves numerous steps and conditional branching, ensuring that each frame is thoroughly analyzed and the extracted embryo images are saved in an appropriate location.

After acquiring the video, the algorithm initializes a variable called folder_counts to one. This variable serves as a counter for the number of folders created during the procedure, which store the segmented embryo images. Following the start of folder_counts, the algorithm sets both start_frame and stop_frame to zero. These two variables are used to manage the range of video frames in the current analysis, with start_frame representing the initial frame to be processed and stop_frame marking the termination of a segment.

Subsequently, the process evaluates the availability of a frame, which is a crucial condition that determines the flow of the algorithm. If this condition is satisfied, there are still video frames to be processed, and the algorithm proceeds to the next step of embryo segmentation. In this phase, the algorithm employs the trained model in the previous step to segment and count embryos within the current frame. However, the process will be completed if the video frame is unavailable.

Upon successful segmentation of embryos, the algorithm evaluates another critical condition: whether the number of segmented embryos surpasses zero. This step serves as a filter to identify frames containing relevant embryonic data. If the algorithm determines that the number of segmented embryos is greater than zero, it implies that the current frame holds valuable information. Consequently, the algorithm proceeds to the next condition, which compares the start_frame value to zero. However, if no segmented embryos exist, the process shifts to the next video frame.

Comparing the start_frame value to zero determines the appropriate course of action in updating the frame range. If the start_frame equates to zero, the algorithm updates the start_frame value and then generates a new folder, incrementing folder_count by a single unit for the next round. This new folder will serve as a repository for storing the segmented embryo images extracted from the subsequent frames. Conversely, if the start_frame value differs from zero, the algorithm updates the stop_frame value, extending the frame range to encompass additional embryonic data.

Subsequently, both scenarios updating start_frame and stop_frame ultimately converge when comparing segmented bounding box coordinates. This step is crucial in ensuring that the extracted embryo images are assigned to their appropriate folders. Notably, each video segment contains the observation of two embryos, which are assigned as left and right based on the x-axis coordinates of their respective segmented bounding boxes. This comparison not only aids in maintaining the organization of the segmented embryo images but also accounts for the relative positioning of the embryos within each frame. Consequently, this method prevents redundancies and ensures that each folder corresponds to a specific embryo while preserving the spatial information of the left and right embryos.

Once the segmented embryo images have been saved in their corresponding folders, the algorithm revisits the initial condition to determine the availability of further frames. This iterative process continues, analyzing each frame and extracting segmented embryo images until no more frames are available for processing. This repetitive approach ensures that the entire video is thoroughly examined and no embryonic data are overlooked or disregarded.

Clear and blurry image classification

**Figure 5:**
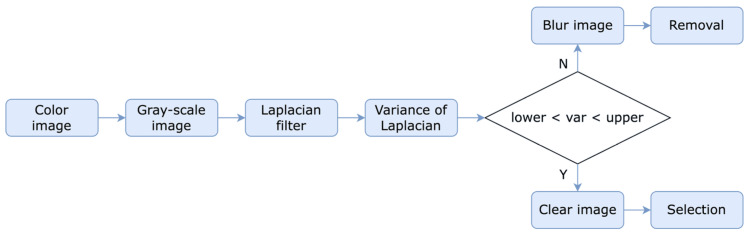
Clear and blurry image classification

The detection of blurred images is a fundamental challenge in the field of image processing and computer vision. The Laplacian filter is a mathematical operator that accentuates image edges and high-frequency components, rendering it a suitable candidate for identifying blurry images. The output of the Laplacian filter is anticipated to have a lower response for a blurred image because the latter typically lacks edges and high-frequency features, which are prevalent in clear images. Figure 5 illustrates a simple image processing algorithm to differentiate between clear and blurry images. The algorithm operates on a color image, converted into a grayscale image to simplify the computation and improve edge detection. The Laplacian filter, a well-known mathematical operator, is then applied to the grayscale image to enhance the edges and high-frequency components of the image, thus facilitating the detection of blurry images. The variance of the Laplacian response is then calculated to determine the degree of variability in the Laplacian response over the entire image. If the variance of the Laplacian response is within a specific range, the image is considered clear, and the algorithm proceeds to the selection step. If the variance of the Laplacian response is outside that range, the image is considered blurry, and the algorithm proceeds to the removal step. The removal step involves removing the blurry image, while the selection step involves selecting a clear image for further processing or analysis.

## Results

Result of embryo segmentation

**Figure 6:**
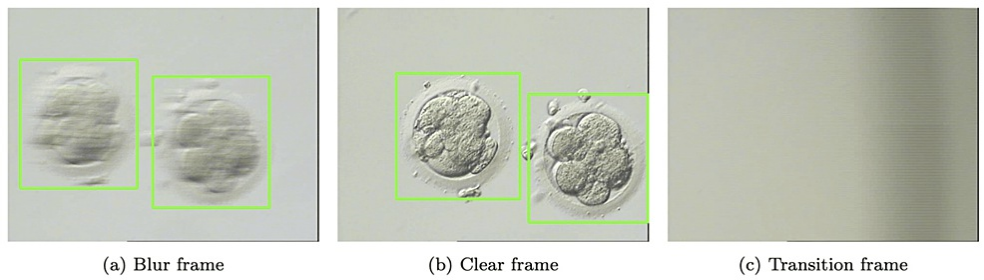
Sample embryonic and transition frames

In this section, we present the results of applying the trained embryo segmentation model to a custom dataset of embryo images, and the outcomes are depicted in Figure 6. Figure 6a showcases a frame that contains two detected blurred embryos. The model accurately segmented the embryos regardless of their low resolution and blurriness. Segmentation outcomes are demonstrated in green bounding boxes. Figure 6b features a frame containing two segmented clear embryos. The frame has a higher quality than the preceding one, and the model produced precise segmentations. This result reveals that the model can handle low- and high-quality images, rendering it very versatile. Lastly, Figure 6c portrays a transition frame that does not encompass any embryo. The model accurately deduced the lack of embryos present and did not produce any segmentation masks. The absence of any segmentation mask in this frame also indicates that the model is very precise and does not produce extraneous output. Furthermore, transitional frames are used to distinguish the observations in each growth medium.

**Figure 7:**
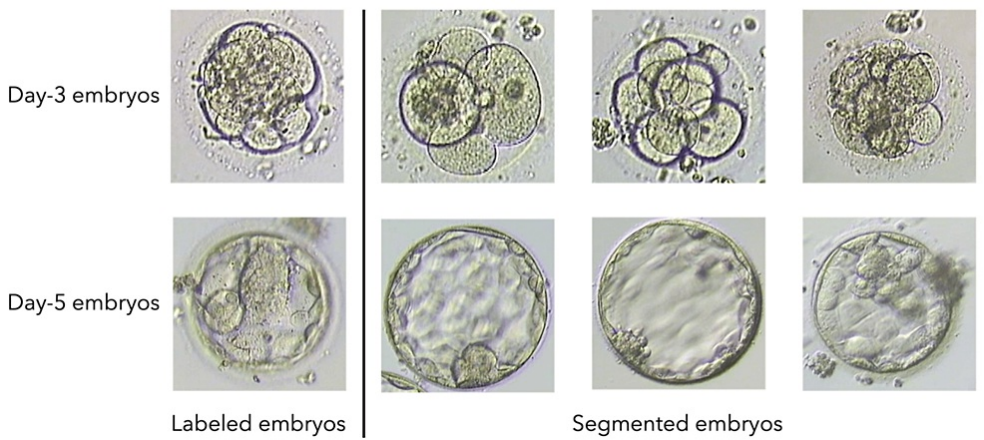
Samples labeled and segmented embryos

Embryos are complex structures, and their morphology changes rapidly during development. This can make it challenging to accurately and consistently label and segment embryos. The YOLO v7 model is capable of detecting and segmenting embryos even when they are at different stages of development or have different sizes, shapes, or orientations. However, the high intra-class variation of the embryos, as presented in Figure 7, causes segmentation challenges. Even within a single species or developmental stage, embryos can exhibit significant morphological variations, making it difficult to identify and segment different structures accurately.

**Figure 8:**
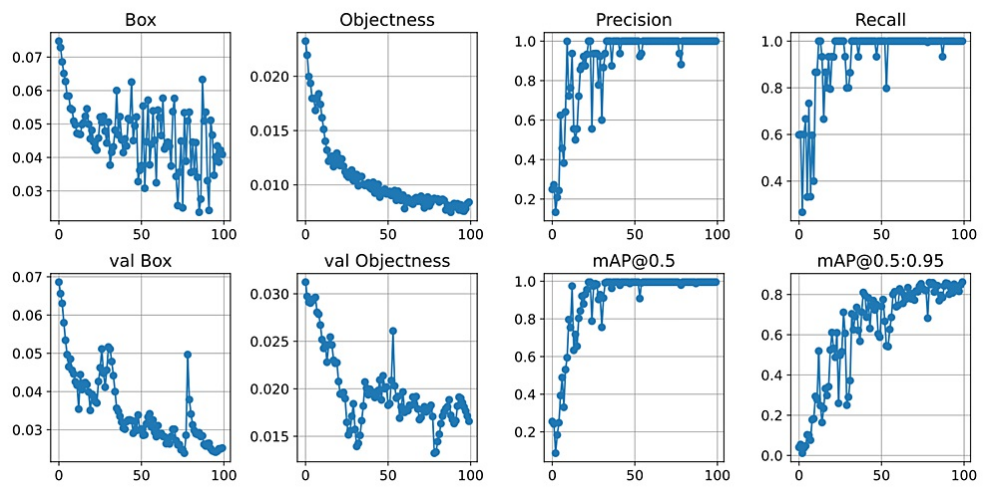
Segmentation result using YOLO v7

Figure 8 shows the segmentation results of the YOLO v7 model on a custom embryo dataset. The box and validation box results provide the localization error, which measures the difference between the predicted position of an object and its ground truth. The box values represent the coordinates of the bounding boxes that the algorithm generates around the detected embryos. In this case, they range from 0.07477 to 0.02362. Similarly, the validation box values range from 0.06861 to 0.02523. A smaller range of values suggests that the algorithm can detect and label embryos across different images consistently.

Based on the objectness and validation objectness results for the YOLO v7 model on the custom embryo dataset, we can observe that the values range from 0.02324 to 0.008406 for the objectness values and 0.03123 to 0.01658 for the validation objectness values. The average validation objectness value is lower than the average, indicating that the algorithm is not performing as well on new and unseen images compared to the training data.

Precision and recall are standard evaluation metrics for object detection models such as YOLO v7. Precision measures the fraction of true positive predictions out of all positive predictions made by the model. In this case, the precision values range from 0.1332 to 1. Higher precision indicates that the model is making fewer false positive predictions. On the other hand, recall measures the fraction of true positive predictions out of all actual positive instances in the dataset. The recall values range from 0.2667 to 1, which indicates that the model is able to detect a larger proportion of positive instances in the dataset. Thus, the model demonstrates a promising result of detecting most of the embryos in the images, and most of the predictions made by the model are correct.

Furthermore, the YOLO v7 model evaluates object detection accuracy using mAP@0.5 and mAP@0.5:0.95. mAP@0.5 represents mean average precision at an IoU threshold of 0.5, while mAP@0.5:0.95. They represent mean average precision across IoU thresholds from 0.5 to 0.95. IoU determines whether detected objects match ground truth objects. The embryo segmentation model has good accuracy with mAP@0.5 values ranging from 0.1332 to 1.0 and mAP@0.5:0.95 values ranging from 0.2667 to 1.0. These results suggest that the model is suitable for practical use in various contexts.

Result of segmented embryo image organization

**Figure 9:**
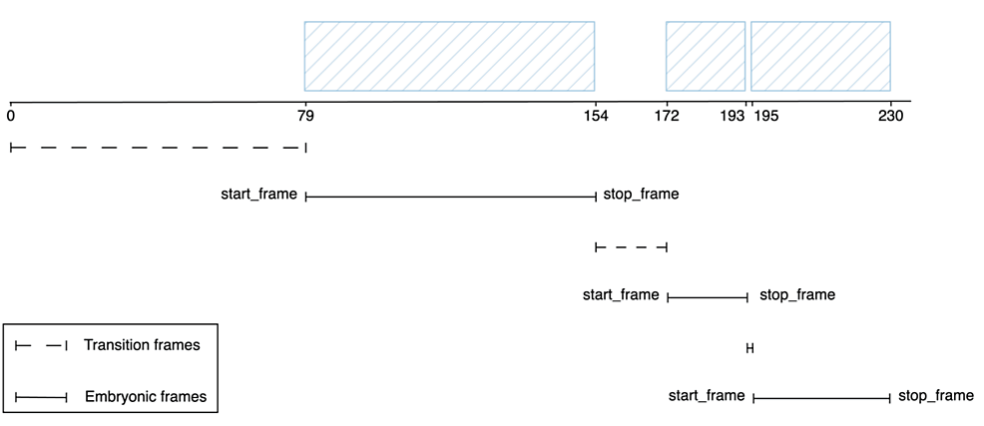
Frame-based embryo segmentation

Frame-based embryo segmentation processing involves dividing the video into distinct frames that aid in identifying and analyzing embryonic development stages, as shown in Figure 9. At the beginning of the session, the embryologists needed time to adjust the microscope for embryonic evaluation. This action resulted in the first segment of frames spanning from frame 0 to frame 79. The embryonic frames of the first growth medium follow in the subsequent segment, from frame 79 to 154, which is approximately 15 seconds long. The embryologist evaluates the first two embryos during this period to determine their development. The next segment, from frames 154 to 172, consists of transition frames that enable the embryologist to move on to the next growth. The second embryonic frame segment spans from frames 172 to 193. It is only four seconds long, indicating that this embryo is not good, and the embryologist need not evaluate its other characteristics. The following segment from frame 963 to 971 contains transition frames that can be shortened by the embryologist’s familiarity with the system. The final segment of embryonic frames covers the third growth medium and lasts 7.2 seconds. In general, bad embryo evaluations take less time than good ones. These time intervals vary depending on the embryologist’s skill and the embryo’s characteristics. The frame-based segmentation process is crucial for identifying and monitoring the embryo development, and it can provide invaluable insights into the mechanisms of embryonic growth and development.

**Figure 10:**
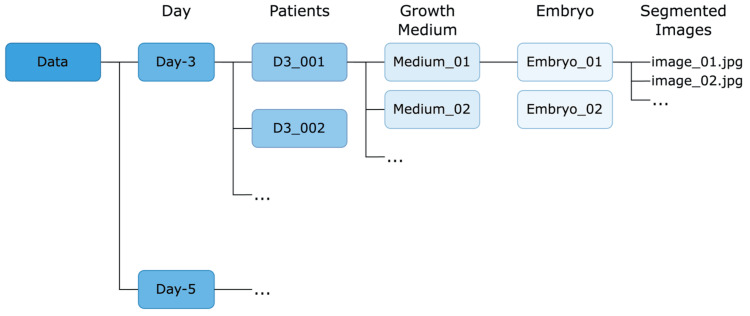
Result of embryo organization

The subsequent step following embryo segmentation is embryo organization. The current research proposes a method for organizing segmented embryo images by using the Day of Development-Patients-Growth Medium-Embryos organizational structure as presented in Figure 10. It enables the effective categorization and storage of segmented images for later training and processing procedures. Grouping the segmented images according to the day, patient, growth medium, and embryo provides a foundation for straightforward comparison and contrast of different embryos’ developmental stages, hence facilitating the identification of possible issues and areas of concern.

At the top level of the organization is the Day of Development variable, which has two subfolders: Day 3 and Day 5. These subfolders represent the time points of embryo development, and each contains the relevant information for all embryos that were observed at that time point. Within each Day-3 folder are multiple subfolders labeled D3_001, D3_002, and so on. These subfolders represent specific patients, and each contains the relevant information for all embryos that were observed for that patient on Day-3. Each patient folder has subfolders for each growth medium used during embryo culture, labeled "Medium_01", "Medium_02", and so on. These subfolders contain the relevant information for all embryos grown in that specific growth medium. Within each growth medium folder, there are two embryo folders labeled "Embryo_01"and "Embryo_02". Each embryo folder contains several images labeled "image_01.jpg", "image_02.jpg", and so on. These images represent selected clear images of the embryo development and provide a clear visual record of the growth and development of each embryo.

This organization enables the straightforward categorization and storage of segmented images for later training and processing procedures. By grouping the segmented images according to the day of development, patient, growth medium, and embryo, it facilitates easy comparison and contrast of different embryos’ developmental stages, identifying potential issues and areas of concern.

Result of clear and blurry image classification

**Figure 11:**
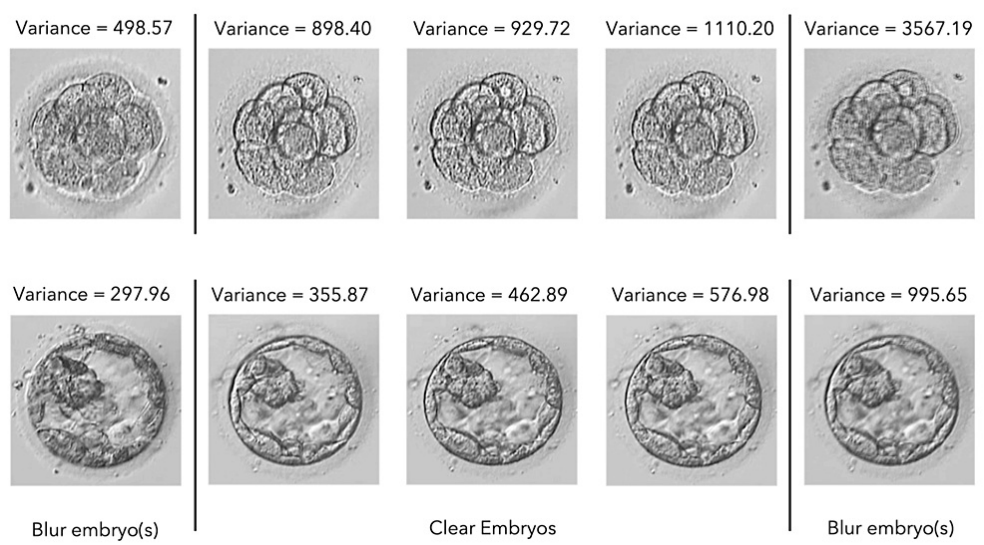
Clear and blurry image examples

Each segmented embryo folder contains both clear and blurry images, which must be differentiated from one another. Detecting clear and blurry images is critical to ensure the performance of the deep learning model in the later stage. The Laplacian filter is a mathematical operator that accentuates image edges and high-frequency components, making it an ideal candidate for detecting blurry images. The Laplacian variance is then calculated to determine the degree of variability in the Laplacian response over the entire image [14]. Figure 11 visualizes the blurred and clear images of day-3 and day-5 embryos. Experimentally, the algorithm sets specific lower and upper boundaries for day-3 and day-5 embryos to determine whether an image is clear or blurry. For day-3 embryos, the lower and upper boundaries are 500 and 2000, respectively, while for day-5 embryos, the lower and upper boundaries are 300 and 900, respectively. If the variance of the Laplacian response falls within the specific range, the image is considered clear, and the algorithm proceeds to the selection step. If the variance of the Laplacian response is outside that range, the image is considered blurry, and the algorithm proceeds to the removal step. The removal step involves removing the blurry image, while the selection step involves selecting a clear image for further processing or analysis.

## Discussion

Integrating AI-based solutions for embryo evaluation is a natural progression in this evolution. However, adopting such solutions depends on various factors, including the IVF center’s existing infrastructure, expertise, and financial resources. For instance, using a conventional inverted microscope offers the benefit of being widely available and relatively cost-effective. However, this approach often requires manual intervention for image capturing, which can be labor-intensive and prone to human error. In this scenario, embryos are evaluated outside the incubator, creating pressure on embryologists to make accurate evaluations quickly. Any delay could affect the embryo’s development due to changes in the surrounding environment. The challenge, therefore, lies in collecting data for AI-based solutions without disrupting the conventional embryo evaluation process. On the other hand, time-lapse technology allows for continuous, automated embryo monitoring within the incubator. This operation provides a dynamic view of the embryo’s development, capturing detailed and temporal valuable information for AI-based analysis. However, the high cost and need for advanced infrastructure make it less accessible, particularly in resource-limited settings. Both scenarios face an added layer of complexity when considering the culturing of grouped embryos, as featured in our manuscript. Grouped embryo culture introduces segmentation and tracking challenges, given the proximity and potential overlap of embryos, thereby demanding more sophisticated AI algorithms for accurate evaluation. This research addresses the use of an inverted microscope for grouped embryo evaluation and reports the first step to establishing an embryo database.

As illustrated in Figure 1, this research confronts a significant challenge when applying an end-to-end segmentation model to videos featuring ’n’ environment drops. The resultant output comprises 2n segmented embryos, all of which are amalgamated into a single folder. This confluence gives rise to a subsequent challenge: the categorization of embryos based on their inherent similarities. The complexity of this issue precludes the possibility of real-time processing for several reasons, including computational limitations, the need for intricate algorithms to distinguish between closely resembling embryos, and the time-intensive nature of validating the accuracy of the segmentation and subsequent grouping.

This research makes several contributions to embryo analysis IVF through DL techniques. One of the most significant achievements is the development of a DL-based model specifically designed to segment embryos precisely. This model addresses the complex challenges associated with segmenting embryos cultured in pairs, a common practice in IVF but a scenario that adds complexity to the segmentation process. Another contribution is introducing an automated system for organizing segmented embryos. This system categorizes embryos based on specific criteria, streamlining the evaluation process and significantly reducing manual labor and the potential for human error. Furthermore, the research introduces an effective classification system that distinguishes clear and blurred embryo images. This classification is automated through DL algorithms, enhancing the system’s efficiency and accuracy. Collectively, these contributions represent a significant advancement in automating and optimizing the process of embryo evaluation in IVF settings.

Another set of pivotal contributions of this research lies in its focus on real-world scenarios, a crucial aspect often overlooked in academic studies. Including varied frame types, such as blurred and transitional frames, in the dataset adds a layer of complexity that closely mimics actual clinical settings. This procedure makes the model more adaptable and robust, capable of handling the intricacies and unpredictabilities commonly encountered in real-world applications. In terms of data collection, the research compiles an extensive and diverse dataset of embryo images and videos. This comprehensive dataset serves as the backbone for training robust DL models and ensures that the results are generalizable and representative of a wide range of scenarios. Furthermore, the dataset is structured to be data-ready for further analysis, providing a valuable resource for future research in this domain. These contributions collectively enhance the study’s relevance and applicability, bridging the gap between academic research and practical clinical needs.

While this research makes several significant contributions, it still has limitations. While the framework developed in this research is designed to be applicable across similar IVF center settings, it is important to note that the actual parameters of the model may require retraining to suit the specific environmental conditions of each center. For instance, variations in imaging equipment, lighting conditions, or even the types of culture media used can impact the model’s performance. Therefore, to adopt this research in a different IVF setting, one would need first to collect a representative dataset from that specific environment. This dataset should then be used to fine-tune the model’s parameters, ensuring that it accurately reflects the unique conditions of the new setting. Additionally, validation studies should be conducted to confirm the model’s efficacy and reliability in the new context. Only after these steps can the model be fully integrated into a different IVF center’s workflow, ensuring accuracy and generalizability.

## Conclusions

In conclusion, the proposed approach of microscopic video-based day-3 and day-5 embryo segmentation using DL is a significant step forward in applying AI in embryo evaluation. The three steps, including the embryo segmentation model, segmented embryo image organization, and clear and blurry image classification, provide an efficient and accurate process for preparing embryo data for further analysis. This research is vital for developing countries seeking to adopt AI in IVF, as it provides a foundation for further analysis and optimization. While there are some limitations to each step, the overall approach is a promising advancement toward a more efficient and accurate analysis of embryo development, ultimately improving the chances of successful IVF outcomes.
